# **Optimal Aminoglycoside Therapy Following the Sepsis: How Much Is Too Much?**

**Published:** 2013

**Authors:** Laleh Mahmoudi, Ramin Niknam, Sarah Mousavi, Arezoo Ahmadi, Hooshyar Honarmand, Shadi Ziaie, Mojtaba Mojtahedzadeh

**Affiliations:** a *Clinical Pharmacy Department, School of Pharmacy, Shiraz University of Medical Sciences, Shiraz, Iran.*; b *Gastroenterohepatology Research Center, Shiraz University of Medical Sciences, Shiraz, Iran.*; c *Gastroenterology and Hepatology Section, Fasa University of Medical Sciences, Fasa, Iran.*; d *Clinical Pharmacy Department, Faculty of Pharmacy, Tehran University of Medical Sciences, Tehran, Iran.*; e *Department of Anesthesiology and Critical Care Medicine, Sina Hospital, Tehran University of Medical Sciences, Tehran, Iran.*; f *Clinical Pharmacy Department, Faculty of Pharmacy, Shahid Beheshti University of Medical Sciences, Tehran, Iran. *

**Keywords:** Sepsis, Septic shock, Aminoglycosides, Pharmacokinetic, Critically ill

## Abstract

Severe sepsis and septic shock are major problems as the result of high rates morbidity and mortality in intensive care units (ICUs). In the presence of septic shock, each hour of delay in the administration of effective antibiotics is associated with a measurable increase in mortality. Aminoglycosides are effective broad-spectrum antibiotics that are commonly used in ICUs for the treatment of life-threatening Gram-negative infections and as a part of empiric therapy for severe sepsis and septic shock. Knowledge of the pharmacokinetic (PK) and pharmacodynamic (PD) properties of the antibiotics used for the management of critically ill patients is essential for selecting the antibiotic dosing regimens and improving patient outcome.

Volume of distribution (Vd) and clearance (CL) of aminoglycosides in critically ill patients differs from general population and these parameters change considerably during the therapy. Pathophysiological changes during the sepsis alter the pharmacokinetic and pharmacodynamic profile of many drugs (increase in Vd and variable changes in CL have been reported for aminoglycosides during the sepsis), therefore, dosing regimen optimization is necessary for achieving therapeutic goal, and critically ill patients should receive larger loading doses of aminoglycosides in order to achieve therapeutic blood levels and due to the considerable variation in kinetic parameters, the use of standard doses of aminoglycosides or dosing nomograms is not recommended in these populations.

## Introduction

Severe sepsis and septic shock are major problems leading to high rates of morbidity and mortality in intensive care units (ICUs) (1, 2). In spite of various medical modalities, mortality due to bacterial infections range from 16% to 50% in sepsis and septic shock respectively (3). 

In the management of sepsis, the role of prompt and appropriate antimicrobial therapy for life-threatening infections is mandatory (4). In the presence of septic shock, each hour delay in the administration of effective antibiotics is associated with a measurable increase in mortality. Kumar, *et al. *(5) showed that effective antimicrobial administration within the first hour of documented hypotension was associated with increased survival in adult patients with septic shock.

Aminoglycoside antibiotics are old, but effective broad-spectrum antibiotics that are commonly used in critical care units for the treatment of life-threatening Gram-negative infections and as a part of empiric therapy for severe sepsis and septic shock, especially if *Pseudomonas aeruginosa *infection is suspected (6). Besides, their use has been associated with less increase in microbial resistance over the years when compared with *β*-lactam antibiotics (7), however, resistance of Gram-negative bacteria to aminoglycosides differs from one region to another and the rate of aminoglycoside resistance is high in some countries (8). Gentamicin, Tobramycin, Amikacin and Netilmicin are the main aminoglycosides still in use.

**Figure 1:**
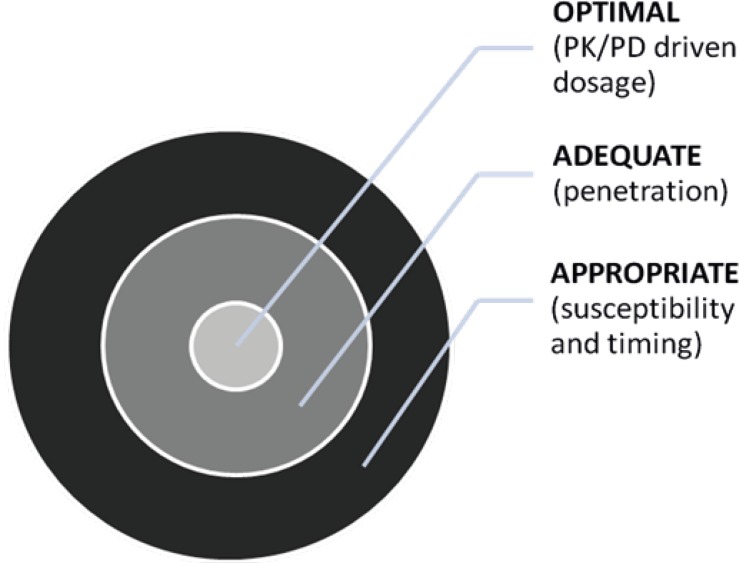
Appropriate, adequate, and optimal antibiotic therapy. Optimal therapy gives the best outcome (9).

Knowledge of the pharmacokinetic (PK) and pharmacodynamic (PD) properties of the antibiotics used for the management of critically ill patients is essential for selecting the antibiotic dosing regimens, which will optimize patient’s outcomes (Figure 1) (9, 10). Thus, we aim to review the effect of septic related changes in critically ill patients with pharmacokinetic parameters, toxicodynamics and optimal dosing of aminoglycosides.

*Pharmacodynamics of aminoglycosides*

Aminoglycosides are hydrophilic antimicrobials that bind to 30S ribosomal A-site RNA, cause the misreading of the genetic code and inhibit the translocation (11). The ensuing antimicrobial activity is usually bactericidal against susceptible aerobic Gram-negative bacilli. Aminoglycosides initially penetrate the organism by disrupting the magnesium bridges between lipopolysaccharide moieties. They are transported across the cytoplasmic membrane in an energy-dependent manner. This step can be inhibited *in-vitro *by divalent cations, increased osmolality, acidic pH, and an anaerobic environment (12).

The Minimum Inhibitory Concentration (MIC) is the lowest concentration of antibiotic that inhibits visible growth of bacteria. The MIC is the most precise method to compare the efficacy of different antibiotics against a particular organism (13, 14), and is reported to the clinician along with the qualitative interpretation (susceptible, intermediate, or resistant).

Aminoglycosides have both a concentration-dependent bactericidal effect and a long post antibiotic effect (PAE) (15). The PAE refers to the persistent suppression of bacterial growth that occurs after the drug has been removed *in-vitro *or cleared by drug metabolism and excretion *in-vivo*. Initially described for Gram-negative bacilli, aminoglycosides also exhibit PAE against *Staphylococcus aureus *but not against other Gram-positive cocci. The duration of the PAE (approximately 3 h; ranges from 1 to 7.5 h) depends upon the method of evaluation and the studied organism (16). PAE characteristic of aminoglycosides may allow sub-MIC trough levels at the end of dosage intervals.

Concentration-dependent killing refers to the ability of higher concentrations of aminoglycosides (relative to the organism’s MIC) to induce more rapid, and complete, killing of the pathogen (17). Because concentration-dependent killing characteristics of aminoglycosides, ratio of the peak drug concentration to the MIC of pathogen or Cmax/MIC is considered to be the parameter that best characterizes the *in-vivo *exposure of the pathogen to serum aminoglycoside concentrations (18, 19). In a retrospective study, Kashuba *et al. *demonstrated that since the peak concentrations and area under the concentration-time curve (AUC) are highly correlated, it has been possible to link both Cmax/MIC and AUC/MIC ratio to outcome. Optimal antibacterial activity is achieved when the peak is 8 to 10 times greater than the MIC (20). To attain effective peak serum concentration and acceptable outcome in critically ill patients, close therapeutic drug monitoring is necessary.

*Pharmacokinetic changes in critically ill patients*

Dosing of aminoglycosides has been discussed in the literature due to the narrow therapeutic index of these agents. Many pathophysiological changes can occur in critically ill patients that can complicate antibiotic dosing. For example, changes in volume of distribution (Vd) and clearance (CL) of antibiotics have been noted in these patients. Initial dosing of drug is determined by Vd, whereas maintenance dosing should be based on CL (21, 22).

Aminoglycosides demonstrate similar PK properties. These agents have similar elimination half-lives of about 2-3 h in normal subjects, and similar Vd (Table 1) (23).

**Table 1:** Pharmacokinetics of aminoglycoside

**Agent**	**Vd**	**Elimination half life Normal**	**ClCr < 10 mL/min**
Amikacin	0.3	2.5-3	30
Gentamicin	0.22-0.3	2.5-3	30-50
Netilmicin	0.26	2-2.3	40
Streptomycin		2.5	100
Tobramycin	0.33	2.5-3	56

Sepsis process is a complex process in which the release of endotoxins (*e.g. *Lipopolysaccharides and lipotechoic acid) and exotoxins from pathogens lead to the production of various endogenous mediators. TNF-α, IL-6 and IL-1*β *are the most important endogenous mediators in the sepsis process, the measurement of which can be helpful in the determination of sepsis severity (2). Release of these mediators can cause endothelial damage and thus increased capillary permeability (24, 25). It is shown that a significant association exists between these biomarker patterns in the first 72 h of sepsis, severity of tissue hypoxia, organ dysfunction, and the mortality (26). This capillary leak leads to the third spacing which is due to the shift of fluid from the intravascular space into the interstitial space. Moreover, the obvious reduction of oncotic pressure resulting from severe hypoalbuminemia (< 1.5 mg/dL) that occurs in some critically ills like head trauma patients, may contribute to the third spacing, which results in the increase of Vd, especially for drugs with small volume of distribution (27-29).

In the Sabzghabaee *et al. *(30) study, the pharmacokinetic behavior of amikacin was evaluated in 31 critically ill patients. In this study, the mean of Vd were 0.39 ± 0.045 (L/Kg) and half of patients had a Vd of more than 0.39 (L/Kg) which is greater than 0.25 L/Kg in comparison with normal population. Resolving of sepsis cause mobilization of excess fluid and reduction in Vd. On the other hand, Vd will be increased proportionally with the increasing levels of sickness severity as measured by the Acute Physiology and Chronic Health Evaluation (APACHE II) score and can result in a decreased *C*_max_ and possibly impairment of their concentration-dependent bactericidal efficacy (31).

Increase Vd of aminoglycosides during sepsis results in subtherapeutic plasma concentrations by using standard dosing regimen (32), even by increasing loading dose of tobramycin and gentamicin from 2 mg/Kg to 3 mg/Kg, only half of the septic patients reach therapeutic C_max_ levels (32, 33).

 Taccone *et al. *(34) assessed the loading dose of amikacin in patients with severe sepsis and septic shock. In this study after a loading dose of 25 mg/Kg (total body weight), the peak concentration remained below therapeutic target level in about one third of these patients, due to the increase in Vd. According to these studies, even by using higher loading dose of aminoglycosides in early phase of sepsis, therapeutic levels can not be achieved in critically ill patients. Subtherapeutic levels increase the risk of multi drug resistance organisms and also mortality rate, therefore, the assessment of blood concentrations and individualization of dosing regimen seems necessary for achieving the therapeutic goal.

It should be considered that beside the capillary permeability, fluid therapy and total parenteral nutrition (TPN) may contribute to expanding the extracellular fluid and Vd and result in the dilution of aminoglycosides in critically ill patients.

In a clinical study, Tormo *et al. *assessed the effect of TPN on kinetic behavior of amikacin in critically ill patients. In this study, 22 critically ill patients enrolled and were treated with 15.5 ± 7.9 mg/Kg/day of amikacin. TPN was administered to malnourished patients while well nourished patients received fluid therapy. An expanded amikacin Vd (0.403 L/Kg vs. 0.298 L/Kg) and a tendency towards increased total body clearance (0.089 L/Kg/h vs. 0.069 L/Kg/h) was shown in TPN group. In addition, TPN produced lower peak concentrations. Tormo *et al *concluded that critically ill patients receiving TPN need higher amikacin doses to ensure the optimal therapeutic response (35). TPN affects gentamicin pharmacokinetic parameters in a similar way (36).

Cardiac output, body weight, oxygen extraction, serum albumin and severity of illness also influence the disposition kinetic of aminoglycosides in severely ill patients with sepsis, so physiologic response to the stress should be considered when determining dosing regimen for critically ill patients (37-40).

Almost all studies showed that the Vd of aminoglycosides was increased in critically ill patients but wide variation in aminoglycosides CL has been reported. Multiple factors such as patient hemodynamic status, vasopressor usage, and cardiopulmonary by-pass may affect the drug›s clearance. Disease states in ICU patients such as burn, trauma and hyperdynamic septic shock increase drug clearance (41).

The body response to infection and injury can cause elevation of metabolic energy expenditure and oxygen consumption and higher cardiac output, blood flow, and organ perfusion. In severe sepsis and septic shock, cardiac index is normal or even increased. In this situation and in the absence of organ dysfunction, renal artery blood flow is also increased that results in the enhanced delivery and excretion of hydrophilic agents like aminoglycosides (42), and consequently their half-life decrease. The clearance of gentamicin was found to be 1.5-fold higher in hyperdynamic septic patients (4.1 ± 0.59 L/min/m²) in comparison with hypodynamic septic patients (2.7 ± 0.43 L/min/m²) (37).

Vasopressor agents that are used for the treatment of hypotension in critically ill patients can cause higher than normal cardiac indices in this population (43, 44). Administration of drugs which influence the glomerular filtration, such as dopamine, dobutamine and diuretics has the same effect (45). Conversely, cardiac failure decreases the renal perfusion and results in reducing the drug clearance and accumulation of aminoglycosides within body, so dosage changing is necessary, but most nomograms use creatinine clearance (CrCl) for the prediction of renal function and dose adjustment. CrCl poorly reflects the changes in renal function in critically ill patients and there is a marked difference between CrCl and total aminoglycosides clearance, thus in this situation, individualized pharmacokinetic dosing is the accurate method for the determination of dosing regimen (46-48).

Among other factors in critically ill patients, mechanical ventilation also affects aminoglycoside kinetic parameters, Vd increase and Cl vary. Tholl *et al. *(49) showed that aminoglycoside clearance rate was 4.37 L/h (0.55-11.56) and the elimination rate constant varied from 0.04 to 0.66/h. It was concluded that the aminoglycoside clearance was highly correlated with physiologic changes in critically ill patients. Aminoglycoside clearance was increased with urinary urea nitrogen excretion and decreased with the increase in blood urea nitrogen concentrations. Besides, they stated that higher albumin and transferrin concentrations were associated with higher aminoglycoside CL rates (50).

The influence of controlled mechanical ventilation on the pharmacokinetic profile of gentamicin in 23 patients after elective open-heart surgery has been reported (38). Lugo *et al. *demonstrated a poor but significant relationship between the application of Positive End-Expiratory Pressure (PEEP) mode (about 10 ± 6 cm H_2_O) and both Vd and clearance of amikacin in 30 critically ill septic patients (39). But in some studies, no inhibitory effects for PEEP were found to increase the concentration (51).

The interpatient variability of aminoglycoside kinetic parameters necessitates dosage individualization of these antimicrobials based on plasma concentrations. Pathophysiological changes in early phase of sepsis necessitate higher loading dose but after resolution of acute phase reevaluation of dosing regimen is crucial for avoiding drug accumulation and toxicity.

*Aminoglycoside toxicity*

Nephrotoxicity develops from aminoglycoside accumulation in the proximal renal tubular cells (52). Polycationic aminoglycosides bind to anionic, brush-border, phospholipid membranes and are transported intracellularly by proximal tubular cells of kidney. Sequestration of these drugs within lysosomes and development of a lysosomal phospholipidosis result in cell necrosis and apoptosis. Tubular necrosis is often accompanied by tubular regeneration and peritubular proliferation; such tubular alterations eventually cause a decline in glomerular filtration and hypo-osmotic polyuria (53, 54). Aminoglycosides’ toxicity is related to the duration of exposure rather than to high serum levels. The kidney is unable to excrete the dose of aminoglycoside within the dosing interval owing to impaired function. Numerous reports evaluate once-daily dosing of aminoglycosides in which the cumulative dose for a 24 h period would be administered as a single dose. This would take advantage of concentration-dependent killing as well as the post-antibiotic effect while minimizing repeated exposure and potential nephrotoxicity. There are differences among the aminoglycosides in terms of renal accumulation and activation of the apoptosis pathway. Gentamicin and Netilmicin have higher renal accumulation compared to Tobramycin and Amikacin (55).

In Nordström *et al. *(56) study, oto- and nephrotoxicity of aminoglycoside were evaluated. Aminoglycosides were given once or three times a day for severe infections. Patients were treated with Netilmicin or gentamicin 4.5 mg/Kg/day, either once a day or divided into three doses. Vestibular function and hearing acuity were examined by serial audiograms and electronystagmogram. The authors concluded that once daily aminoglycoside treatment has not greater oto- or nephrotoxicity compared to the conventional three times daily regimen.

However, Olsen *et al. *(57) showed that the once daily dose tobramycin regimen appeared to be less nephrotoxic in comparison with the multiple daily dose regimens despite significantly higher doses. In this study, the Tobramycin dose was higher in the once daily dose group in comparison with the multiple daily dose group (425 ± 122.5 mg vs. 312.8 ± 116.6 mg, p < 0.001). In the once daily dose group, patients had a higher measured creatinine clearance at the end of therapy compared with multiple daily dose group (70 ± 18.6 vs. 64.8 ± 17.5 mL/min, p = 0.047). Fewer patients in the once daily dose group developed nephrotoxicity than the multiple daily dose group (5 vs. 12, p = 0.142). Although there were increases in urinary enzymes in both treatment groups (alanine aminopeptidase, 8.7 ± 2.9 vs. 5.2 ± 2.1 units/24 h, p < 0.01 multiple daily dose vs. once daily dose; *n*-acetyl-beta-d-glucosaminidase, 14.7 ± 4.9 vs. 6.8 ± 3.1, p < 0.01 multiple daily dose vs. once daily dose), increases in the once daily dose group were significantly lower than that of multiple daily dose group. Olsen *et al. *stated that Tobramycin administered by once daily dose may be the preferred dosing method in selected critically ill medical patients to reduce the incidence and extent of renal damage.

Aminoglycoside exposure-nephrotoxicity relationship was observed in randomized, double blind comparison of once- vs. twice-daily dosing of aminoglycosides (58, 59). There was a major shift in the probability of nephrotoxicity from once-daily to twice-daily dosing. Time to the occurrence of a nephrotoxic event was a function of days of therapy, stratified by whether patients received vancomycin concurrently. In the absence of concurrent vancomycin use, there was a very small probability of a nephrotoxic event at day 7. In contrast, the likelihood of nephrotoxicity was exacerbated by concurrent administration of vancomycin. The once-daily dosing group had a low probability of nephrotoxicity even with the co-administration of vancomycin. Consequently, by administering the drug once daily, avoiding concurrent nephrotoxins and limiting duration of therapy to 7 days, these agents can be safely administered in the ICU setting.

The relationship between ototoxicity and aminoglycoside pharmacokinetic is not clear, but it can be expected that in duration less than 5-6 days, its incidence is low and this side effect is seen in prolonged therapy for 10 days or more.

It can be said that once-daily dosing regimens of aminoglycosides have less nephrotoxicity compared to the multiple-daily dosing, although once-daily dosing use for long time (> 7 days) can result in substantial nephrotoxicity (Table 2). Aminoglycoside dosing considerations in critically ill patients should attempt to improve the outcome meanwhile reducing the toxicity.

**Table 2:** Recommended dosing schedules for adult patients with impaired renal function (60).

**Estimated Creatinine Clearance (mL/min)**	**Dose (mg/Kg** **)**		**Dosing Interval (h)**
	**Gentamicin, Tobramycin**	**Amikacin, Netilmicin**	
100	7	20	24
90	7	20	24
80	7	20	24
70	5	15	24
60	5	15	24
50	4	12	24
40	4	12	24
30	5	15	48
20	4	12	48
10	3	10	48
> 10	2.5	7.5	48

## Conclusion

Although antibiotic therapy is cornerstone modality in sepsis treatment, due to the pathophysiological changes during this process, optimal dosing of antibiotics is a major challenge in critical care for better outcome. However, owing to the clinically significant variations in aminoglycoside pharmacokinetics, routine use of conventional aminoglycoside dosing nomograms to achieve an appropriate dosage regimen is discouraged in many critically ill patients. The aminoglycoside volume of distribution (Vd) and clearance (CL) in critically ill patients differs from general population and pharmacokinetic parameters change considerably during the therapy. Critically ill patients should undergo individualized pharmacokinetic analysis to characterize efficiently and more effectively plasma concentrations and determine an appropriate dosing interval, considering site and severity of infection, plasma clearance, and the apparent post-antibiotic effect. Based on these data, the majority of critically ill patients would not be predicted to achieve the PD target under current dosing regimens. Since critically ill patients have significantly larger Vd and altered CL, they may require larger doses per kilogram of body weight of aminoglycoside and should receive larger loading doses of aminoglycosides in order to achieve therapeutic blood levels. Besides, the use of extended-interval aminoglycoside dosing regimens should be based on pharmacodynamic endpoints and patient-specific pharmacokinetic assessment.

## References

[1] Panahi Y, Mojtahedzadeh M, Beiraghdar F, Pazooki M, Moharamzad Y (2008). Prevalence of Microorganisms Causing Septicemia and Determination of Antimicrobial Resistance in Intensive Care Unit. Iranian J. Pharm. Res.

[2] Hamishehkar H, Beigmohammadi MT, Abdollahi M, Ahmadi A, Mahmoodpour A, Mirjalili MR, Abrishami R, Khoshayand MR, Eslami K, Kanani M, Baeeri M, Mojtahedzadeh M (2010). Identification of enhanced cytokine generation following sepsis. Dream of magic bullet for mortality prediction and therapeutic evaluation. Daru.

[3] Rafati MR, Rouini MR, Mojtahedzadeh M, Najafi A, Tavakoli H, Gholami K, Fazeli MR (2006). Clinical efficacy of continuous infusion of piperacillin compared with intermittent dosing in septic critically ill patients. Intern. J. Antimicrob. Agent.

[4] Dellinger RP, Levy MM, Carlet JM (2008). Surviving Sepsis Campaign: international guidelines for management of severe sepsis and septic shock. Crit. Care Med.

[5] Kumar A, Roberts D, Wood KE, Light B, Parrillo JE, Sharma S, Suppes R, Feinstein D, Zanotti S, Taiberg L, Gurka D, Kumar A, Cheang M (2006). Duration of hypotension prior to initiation of effective antimicrobial therapy is the critical determinant of survival in human septic shock. Crit. Care Med.

[6] Falagas ME, Kopterides P (2007). Old antibiotics for infections in critically ill patients. Curr. Opin. Crit. Care.

[7] Roberts JA, Lipman J (2009). Pharmacokinetic issues for antibiotics in the critically ill patients. Crit. Care. Med.

[8] Mojtahedzadeh M, Panahi Y, Fazeli MR, Najafi A, Pazouki M, Mahdi-Navehsi B, Bazzaz A, Naghizadeh MM, Beiraghdar F (2008). Intensive care unit-acquired urinary tract infections in patients admitted with sepsis: etiology, risk factors, and patterns of antimicrobial resistance. Int. J. Infect. Dis.

[9] Nicolau DP (1998). Optimizing antimicrobial therapy and emerging pathogens. Am. J. Managed. Care.

[10] Ulldemolins M, Nuvials X, Palomar M, Masclans JR, Rello J (2011). Appropriateness is Critical. Crit. Car. Clin.

[11] Fourmy D, Recht MI, Blanchard SC, Puglisi JD (1996). Structure of the A site of Escherichia coli 16S ribosomal RNA complexes with an aminoglycoside antibiotic. Science.

[12] Mingeot-Leclercq MP, Glupczynski Y, Tulkens PM (1999). Aminoglycosides: Activity and resistance. Antimicrob Agents Chemother.

[13] Royo P, Martin-Casabona N, Martinez E, Andonegui M (1999). In-vitro susceptibility of Mycobacterium kansasii to the difluorinated quinolone sparfloxacin using a broth microdilution and macrodilution MIC system. Int. J. Tuberc. Lung. Dis.

[14] Jorgensen JH, Ferraro MJ (1998). Antimicrobial susceptibility testing: general principles and contemporary practices. Clin. Infect. Dis.

[15] Fisman DN, Kaye KM (2000). Once-daily dosing of aminoglycoside antibiotics. Infect. Dis. Clin. North Am.

[16] Novelli A, Mazzei T, Fallani S, Cassetta MI, Conti S (1995). In-vitro postantibiotic effect and postantibiotic leukocyte enhancement of tobramycin. J. Chemother.

[17] McLean AJ, Ioannides-Demos LL, Li SC, Bastone EB, Spicer WJ (1993). Bactericidal effect of gentamicin peak concentration provides a rationale for administration of bolus doses. J. Antimicrob. Chemother.

[18] Moore RD, Lietman PS, Smith CR (1987). Clinical response to aminoglycoside therapy: importance of the ratio of peak concentration to minimal inhibitory concentration. J Infect. Dis.

[19] Deziel-Evans LM, Murphy JE, Job ML (1986). Correlation of pharmacokinetic indices with therapeutic outcome in patients receiving aminoglycosides. Clin. Pharm.

[20] Kashuba AD, Nafziger AN, Drusano GL, Bertino JS (1999). Optimizing aminoglycoside therapy for nosocomial pneumonia caused by gram-negative bacteria. Antimicrob. Agents Chemother.

[21] Nicolau DP (2003). Optimizing outcomes with antimicrobial therapy through pharmacodynamic profiling. J. Infect. Chemother.

[22] Roberts JA, Kruger P, Paterson DL, Lipman J (2008). Antibiotic resistance - what’s dosing got to do with it?. Crit. Care. Med.

[23] Turnidge J (2003). Pharmacodynamic and dosing of aminoglycosides. Infect. Dis. Clin. North Am.

[24] Bochud PY, Calandra T (2003). Pathogenesis of sepsis: new concepts and implications for future treatment. BMJ.

[25] Glauser MP, Zanetti G, Baumgartner JD, Cohen J (1991). Septic shock: pathogenesis. Lancet.

[26] Mahmoodpoor A, Eslami K, Mojtahedzadeh M, Najafi A, Ahmadi A, Dehnadi-Moghadam A, Mohammadirad A, Baeeri M, Abdollahi M (2010). Examination of Setarud (IMOD™) in the management of patients with severe sepsis. Daru.

[27] Shohrati M, Mojtahedzadeh M, Rouini MR, Gholami K, Eftekhar B, Sadidi A, Abdollahzadeh M (2002). Correlation of free fraction of phenytoin and plasma albumin level in head trauma patients. Daru.

[28] Joynt GM, Lipman J, Gomersall CD, Younga RJ, Wonga ELY, Gina T (2001). The pharmacokinetics of once-daily dosing of ceftriaxone in critically ill patients. J. Antimicrob Chemother.

[29] Vrhovac B, Sarapa N, Bakran I, Huic M, Macolic-Sarinic V, Francetic I, Wolf-Coporda A, Plavsic F (1995). Pharmacokinetic changes in patients with edema. Clin. Pharmacokinet.

[30] Sabzghabaee AM, Mojtahedzadeh M, Tajerzadeh H, Asasi N, Ganji M, Mohagheghi A, Gholami K, Hadavand N (2002). Pharmacokinetic behavior of amikacin in 31 iranian critically ill septic patients. Daru.

[31] Marik PE (1993). Aminoglycoside volume of distribution and illness severity in critically ill septic patients. Anaesthesia Intens. Care.

[32] De Paepe P, Belpaire FM, Buylaert WA (2002). Pharmacokinetic and pharmacodynamic considerations when treating patients with sepsis and septic shock. Clin. Pharmacokinet.

[33] Dorman T, Swoboda S, Zarfeshenfard F, Trentler B, Lipsett PA (1998). Impact of altered aminoglycoside volume of distribution on the adequacy of a three milligram per kilogram loading dose. Surgery.

[34] Taccone F, Laterre PF, Spapen H, Dugernier T, Delattre I, Layeux B (2010). Revisiting the loading dose of amikacin for patients with severe sepsis and septic shock. Crit. Care.

[35] Tormo C, Abad FJ, Ronchera-Oms CL, Parra V, Jimenez NV (1995). Critically-ill patients receiving total parentral nutrition show altered amikacin pharmacokinetics. Clin. Nutr.

[36] Ronchera-Oms CL, Tormo C, Ordovás JP, Abad J, Jiménez NV (1995). Expanded gentamicin volume of distribution in critically ill adult patients receiving total parenteral nutrition. J. Clin. Pharm. Ther.

[37] Triginer C, Izquierdo I, Fernandez R, Rello J, Torrent J, Benito S (1990). Gentamicin volume of distribution in critically ill septic patients. Intens. Care. Med.

[38] Lugo G, Castaneda-Hernandez G (1997). Relationship between hemodynamic and vital support measures and pharmacokinetic variability of amikacin in critically ill patients with sepsis. Crit. Care. Med.

[39] Hassan E, Ober JD (1987). Predicted and measured aminoglycoside pharmacokinetic parameters in critically ill patients. Antimicrob. Agents. Chemother.

[40] Tholl DA, Shikuma LR, Miller TQ, Woodward J, Cerra FB, Zaske DE (1993). Physiologic response of stress and aminoglycoside clearance in critically ill patients. Crit. Care. Med.

[41] Hadavand N, Mojtahedzadeh M, Sadray S, Shariat Moharreri R, Shafaghic B, Khajavi MR, Salari P (2004). Pharmacokinetic Behavior of Theophylline following PEEP in Critically Ill Patients with Acute Lung Injury. Iranian J. Pharm. Res.

[42] Pea F, Viale P (2006). The antimicrobial therapy puzzle: Could pharmacokinetics-pharmacodynamics relationships be helpful in addressing the issue of appropriate pneumonia treatment in critically ill patients? Clin. Infect. Dis.

[43] Parrillo JE (1993). Pathogenetic mechanisms of septic shock. NEJM.

[44] Pea F, Porreca L, Baraldo M, Furlanut M (2000). High vancomycin dosage regimens required by intensive care unit patients cotreated with drugs to improve haemodynamics following cardiac surgical procedures. J. Antimicrob. Chemother.

[45] Scaglione F, Paraboni C (2008). Pharmacokinetics/ pharmacodynamics of antibacterials in the intensive care unit: setting appropriate dosing regimens. Int. J. Antimicrob. Agents.

[46] Hickling KG, Begg EJ, Perry RE, Atkinson HC, Sharman JR (1991). Serum aminoglycoside clearance is predicted as poorly by renal aminoglycoside clearance as by creatinine clearance in critically ill patients. Crit. Care. Med.

[47] Lugo-Goytia G, Castaeda-Hernbndez G (2000). Bayesian approach to control of amikacin serum concentrations in critically ill patients with sepsis. Annal. Pharmacother.

[48] Kashuba AD, Bertino Jr JS, Nafziger AN (1998). Dosing of aminoglycosides to rapidly attain pharmacodynamic goals and hasten therapeutic response by using individualized pharmacokinetic monitoring of patients with pneumonia caused by gram-negative organisms. Antimicrob. Agents Chemother.

[49] Tholl DA, Shikuma LR, Miller TQ, Woodward JM, Cerra FB, Zaske DE (1993). Physiologic response of stress and aminoglycoside clearance in critically ill patients. Crit. Care. Med.

[50] Conil JM, Georges B, Lavit M, Laguerre J, Samii K, Houin G, Saivin (2007). A population pharmacokinetic approach to caftazidime use in burn patients: Influence of glomerular filtration, gender and mechanical ventilation. Br. J. Clin. Pharmacol.

[51] Hadidi E, Mojtahedzadeh M, Rouini MR, Eftekhar B, Abdollahi M, Najafi A, Khajavi MR, Rezaee S, Ghaffari R, Afshar M (2005). The evaluation of the possible effect of positive end expiratory pressure (PEEP) on pharmacokinetics of phenytoin in patients with acute brain injury under mechanical ventilation. Daru.

[52] Giuliano RA, Verpooten GA, Verbist L, Wedeen RP, De Broe ME (1986). In-vivo uptake kinetics of aminoglycosides in the kidney cortex of rats. J. Pharmacol. Exp. Ther.

[53] Tulkens PM (1989). Nephrotoxicity of aminoglycoside antibiotics. Toxicology Letters.

[54] Swan SK (1997). Aminoglycoside nephrotoxicity. Seminars in Nephrology.

[55] Winslade NE, Adelman MH, Evans ES, Schentag JJ (1987). Single dose accumulation kinetics of tobramycin and netilmicin in normal volunteers. Antimicrob. Agents Chemother.

[56] Nordström L, Ringberg H, Cronberg S, Tjernström O, Walder M (1999). Does administration of an aminoglycoside in a single daily dose affect its efficacy and toxicity? J. Antimicrob. Chemother.

[57] Olsen KM, Rudis MI, Rebuck JA, Hara J, Gelmont D, Mehdian R, Nelson C, Rupp ME (2004). Effect of once-daily dosing vs. multiple daily dosing of tobramycin on enzyme markers of nephrotoxicity. Crit. Care. Med.

[58] Drusano GL, Ambrose PG, Bhavnani SM, Bertino JS, Nafziger AN, Louie A (2007). Back to the future: using aminoglycosides again and how to dose them optimally. Clin. Infect. Dis.

[59] Rybak MJ, Abate BJ, Kang SL, Ruffing MJ, Lerner SA, Drusano GL (1999). Prospective evaluation of the effect of an aminoglycoside dosing regimen on rates of observed nephrotoxicity and ototoxicity. Antimicrob. Agent. Chemother.

[60] Craig WA (2011). Optimizing Aminoglycoside Use. Crit. Care. Clin.

